# Sclerosing epithelioid fibrosarcoma: rare and serious

**DOI:** 10.11604/pamj.2020.36.131.18668

**Published:** 2020-06-25

**Authors:** Mounir Yahyaoui, Mohammed Benhammou, Soufiane Aharram, Jawad Amghar, Mohammed Sadougui, Omar Agoumi, Abdelkrim Daoudi

**Affiliations:** 1 Trauma-Orthopedics Service, University Hospital Mohammed 6th Oujda, Oujda, Morocco

**Keywords:** Sclerosing epithelioid fibrosarcoma, surgery, adjuvant treatment, recurrences, metastases

## Abstract

Sclerosing Epithelioid Fibrosarcoma (SEF) is a rare form of soft tissue sarcoma. It is characterized by a slow evolution, with local recurrences and late metastases that are mainly pulmonary and pleural in about 50% of cases. The treatment is based on the surgery which must be as wide as possible. The efficacy of adjuvant therapy in the control of SEF is not yet demonstrated. Chemotherapy is used in recurrences, some have also proposed radiotherapy. Long-term follow-up of patients with SEF is therefore essential.

## Introduction

Enzinger and Weiss were the first in 1988 to describe a variant of fibrosarcoma with a predominance of epithelioid cells [1]. In 1995, Meis-Kindblom *et al*. reported a series of 25 cases and introduced the term “sclerosing epithelioid fibrosarcoma” (SEF) [2]. This new histological entity is distinguished from other fibrosarcomas by slow growth. Histological diagnosis can be difficult, and the differential diagnosis should be discussed with benign soft tissue tumors, metastatic carcinomas, and high grade sarcomas. Of the 50 cases reported in the literature, local recurrences and distant metastases have been described [2, 3]. Metastases are rare and usually occur several years after the initial surgical treatment.

## Patient and observation

**Figure 1:**
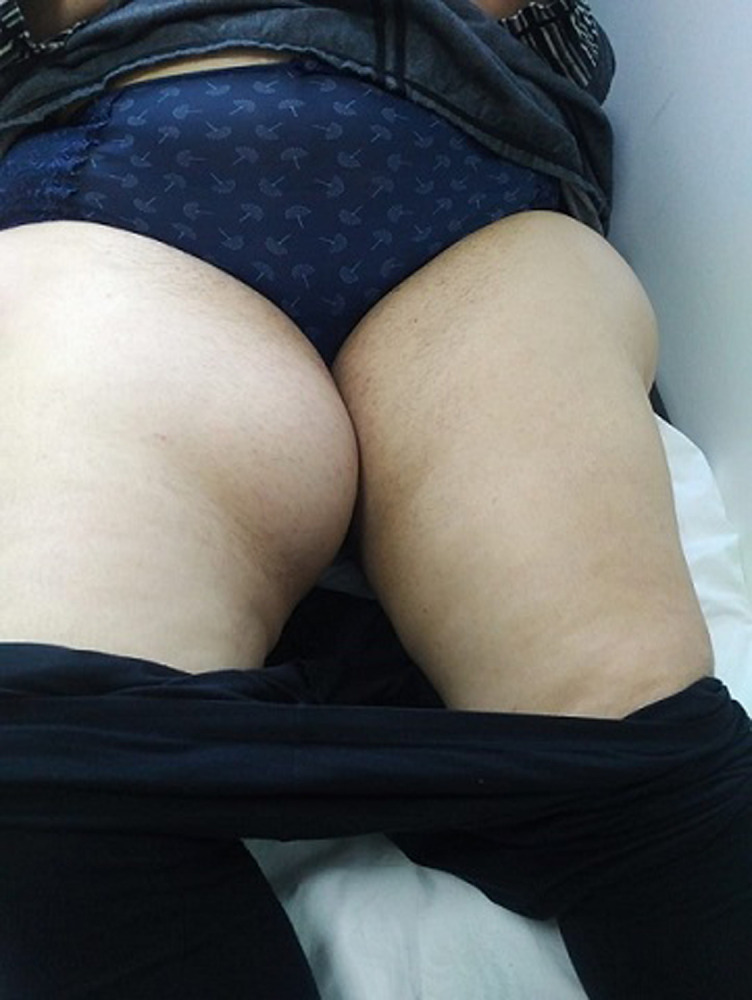
clinical aspect of the mass

**Figure 2:**
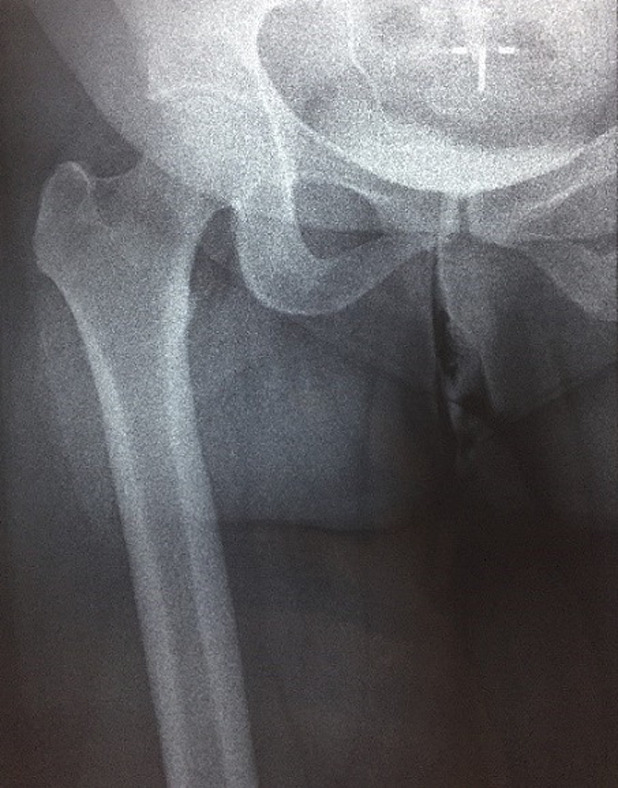
X-ray of the thigh

**Figure 3:**
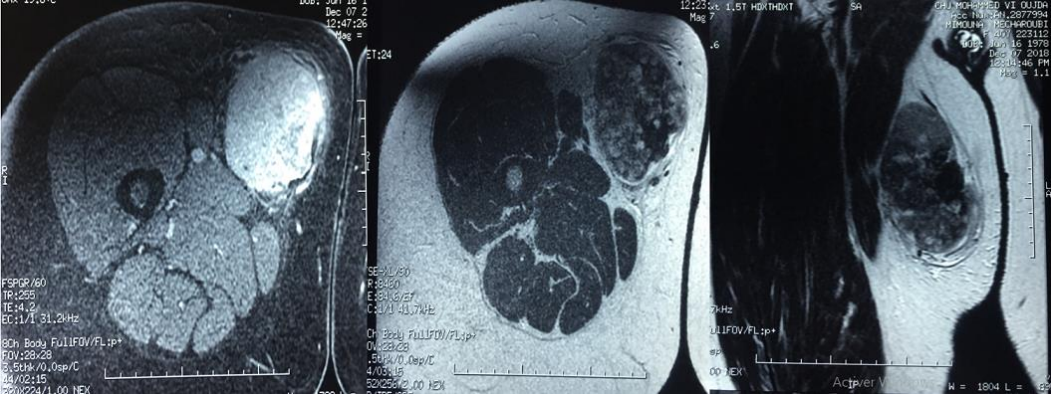
MRI appearance of the mass

**Figure 4:**
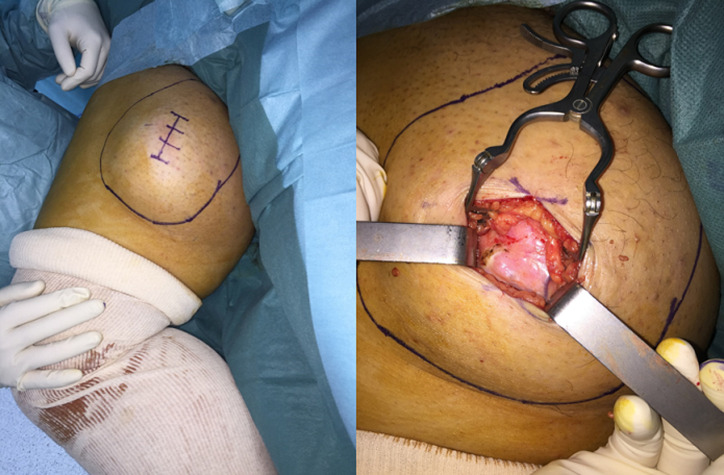
biopsy first

**Figure 5:**
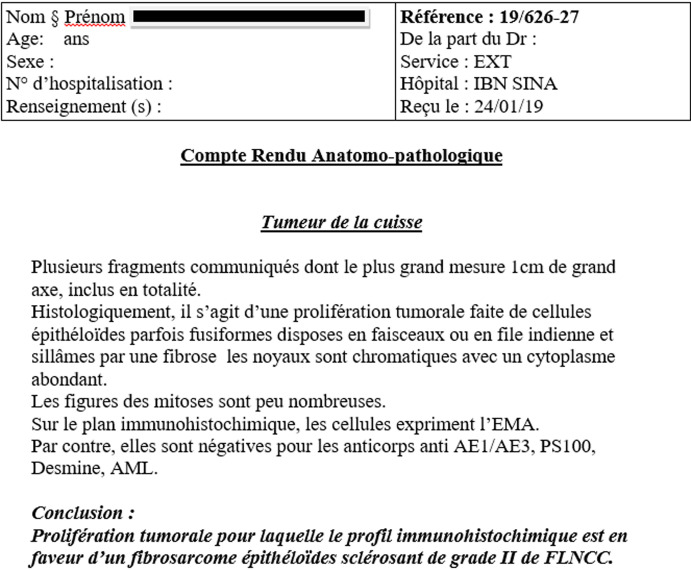
anatomopathological result

**Figure 6:**
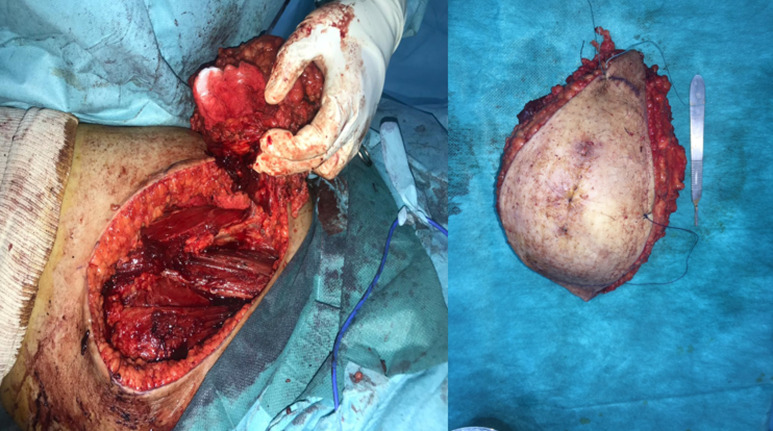
wide excision of the tumor

M.M female, had for 20 years a mass of the inner side of the right thigh painless, gradually increasing in volume, and evolving in a context of apyrexia and conservation of the general state. The clinical examination showed a mass at the antero-internal surface of the right thigh (Figure 1) of 18/12cm, well limited, firm consistency, non-flapping, painless on palpation, mobile in relation to the superficial plane and adherent to the deep one, with no inflammatory signs or satellite lymphadenopathies with a normal neurovascular examination. The patient underwent standard radiography of the thigh (Figure 2) revealing opacity of the soft tissues, and MRI (Figure 3) which showed a sub fascial mass of the inner side of the right thigh, well-defined, contours irregular in places, in heterogeneous hypersignal T2/isosignal T1, closing areas in hyposignal T1 and T2 in relation to calcifications, and raising intensively and heterogeneously after injection of contrast product. This mass drove the superficial veins and respected the neighboring muscular structures. She first benefited from a surgical biopsy with regard to the mass (Figure 4) whose pathology showed epithelioid cells sometimes fusiform arranged in bundles or single file and marked by fibrosis, the nuclei were chromatic with abundant cytoplasm, the figures of the mitoses were few. Immunohistochemically, cells expressed EMA, but were negative for AE1/AE3, PS100, Desmin, AML. It is therefore a FLNCC grade II of sclerosing epithelioid fibrosarcoma (Figure 5). The extension assessment was negative (thoraco-abdominopelvic computed tomography (CT) and bone scintigraphy), and after a multidisciplinary consultation meeting, the decision was a first surgery. A large excision was made taking the path of the biopsy including without seeing the tumor with a margin of safety of 4cm reaching the superficial plane of sartorius (Figure 6). The anatomopathological result confirmed the same diagnosis with resection margins at R0. The patient is a candidate for adjuvant radiotherapy.

## Discussion

Sarcomas are malignant tumors with mesenchymal differentiation. Fibrosarcoma accounts for 1 to 2% of all sarcomas [4]. There are several variants, including sclerosing epithelioid fibrosarcoma, which is a rare variant of poor prognosis [5]. It is composed of epithelioid cells arranged in cell clusters or cords in an abundant hyalinized collagenic stroma [6]. Among the 50 cases reported in the literature [2, 3], there were 26 women for 24 men, with an average age of 45 years (range: 14-87 years); only 3 patients were under 20 years old. Delayed diagnosis is common in non-specific symptoms such as trailing pain. SEF can reach all parts of the body, but with preferential localization for the proximal limb, trunk, head and neck [2, 3]. SEF is considered a low grade sarcoma. Local recurrences and metastases are not uncommon. The local recurrence rate is about 50%, with an average delay of 3.5 years (range: 2 months-11 years). Metastases occur in 43 to 86% of cases, with a delay of 3 to 14 years [2, 3]. They are localized preferably in the lungs, the pleura and the bone. The diagnostic and therapeutic strategy of soft tissue tumors is currently well codified. It depends on the size and superficial or deep nature of the tumor. Biopsy excision of a small soft tissue tumor as soon as it is superficial may be considered. If the tumor is large as in our case, a locoregional extension assessment must be performed before any surgical procedure. It is essentially based on an MRI that can guide the biopsy and plan surgical resection. The preliminary surgical biopsy, which must be performed without risk of dissemination, establishes the benign or malignant nature of the tumor. In addition, it makes possible, as far as possible, typing and histological grading for sarcomas [7, 8]. The treatment is based on surgery which must be as wide as possible [9]. The efficacy of adjuvant therapy in the control of SEF is not yet demonstrated [10]. Chemotherapy is used in cases of recurrence based on a combination of several molecules including adriamycin and ifosfamide [11-13]. Some also proposed radiotherapy [13]. Regular and long-term follow-up is recommended since recurrences or distant metastases can be seen late [2].

## Conclusion

SEF is a rare form of soft tissue sarcoma. It is characterized by a slow evolution, with local recurrences and late metastases that are mainly pulmonary and pleural in about 50% of cases. Long-term follow-up of patients with SEF is therefore essential.
